# Feasibility of remote assessment of the binaural intelligibility level difference in school-age children

**DOI:** 10.1121/10.0003323

**Published:** 2021-01

**Authors:** Gabrielle R. Merchant, Claire Dorey, Heather L. Porter, Emily Buss, Lori J. Leibold

**Affiliations:** 1 Boys Town National Research Hospital, Center for Hearing Research, Omaha, Nebraska 68131, USA; 2 Department of Speech, Language, and Hearing Sciences, University of Florida, Gainesville, Florida 32610, USA; 3 Department of Otolaryngology/HNS, University of North Carolina at Chapel Hill, Chapel Hill, North Carolina, USA Gabrielle.Merchant@boystown.org, cdorey@ufl.edu, Heather.Porter@boystown.org, ebuss@med.unc.edu, Lori.Leibold@boystown.org

## Abstract

This work evaluated the feasibility and reliability of remotely assessing masked speech recognition and the binaural intelligibility level difference (BILD) in children. Participants were 28 children (6–17 years) and 11 adults (22–45 years) with self-reported normal hearing. A three-alternative forced-choice word recognition task was completed using participants' personal hardware (headphones and computer) and custom software that uploaded results to a central database. Results demonstrate that assessment of masked speech recognition and the BILD is feasible and generally reliable in a remote setting. Variability of results across individuals would likely have been reduced by distributing or specifying appropriate headphones.

## 1. Introduction

The purpose of this study was to examine the feasibility and reliability of a three-alternative forced-choice (3AFC) word recognition procedure for remote assessment of the binaural intelligibility level difference (BILD) in children. The BILD refers to the improvement in masked speech recognition threshold (SRT) typically observed in a diotic masker when target speech is presented 180° out-of-phase to one ear (*M*_0_*T*_π_) relative to when target speech is presented in-phase across the two ears (*M*_0_*T*_0_). A BILD of 5–8 dB has been reported for adults with normal hearing when target words are presented in a broadband noise masker (e.g., Licklider 1948; Johansson and Arlinger, 2002; Goverts and Houtgast, 2010).

Although there are not many studies of the BILD in children, the data that are available indicate that school-age children benefit in the *M*_0_*T*_π_ condition, and the BILD increases with increasing age. For example, Koopmans *et al.* (2018) evaluated the BILD in a group of 112 children, 4–12 years of age, and 33 adults, all with normal hearing. Targets were three-digit numbers and maskers were steady noise. SRTs improved with increasing child age in both the M_0_T_0_ and M_0_T_π_ conditions, and the mean BILD increased from 3 dB for 4- to 6-year-olds to 5 dB for adults. Analogous developmental effects have been reported for the binaural masking level difference (MLD), a related paradigm using a pure-tone target and a target detection task (e.g., Hall and Grose, 1990; Hall *et al.*, 1995). Data indicate a positive MLD for children as young as 4 to 5 years of age, although this effect is not as large as observed for adults (e.g., Hall and Grose 1990). Compared to the MLD, the BILD may be a more promising method for evaluating binaural hearing in children because speech stimuli might maintain a child's interest longer than pure tones, can be illustrated and incorporated into a picture-pointing task, and have greater ecological validity than pure tones.

One possible application for a BILD-based assessment is to monitor binaural hearing abilities in children with chronic otitis media with effusion (OME). Several laboratories have shown that children with a history of chronic OME have significantly lower MLDs than their age-matched peers with no history of ear disease (e.g., Moore *et al.*, 1991; Hall and Grose, 1993). Importantly, these binaural hearing deficits can persist for up to two years following corrective surgery (Hall *et al.*, 1995). Performance on an antiphasic digits-in-noise test has also been demonstrated to be sensitive to both sensorineural and conductive hearing loss (De Sousa *et al.*, 2020). Implementing a convenient and simple approach for remote estimation of the BILD could provide an opportunity to track performance over time in children with active and/or resolved OME.

Although recent interest in remote testing has been largely driven by public health concerns surrounding COVID-19, the potential benefits of remote testing for speech perception and psychophysical experiments extend beyond the pandemic (e.g., de Graaff *et al.*, 2019; Woods *et al.*, 2017). Remote testing may reduce barriers for participation in research (e.g., travel to the laboratory, testing during business hours), providing an opportunity to recruit a larger and more diverse participant sample (e.g., Rezlescu *et al.*, 2020). While few published studies report using remote testing methods to evaluate children's auditory abilities (e.g., Rashid *et al.*, 2016), additional advantages for use with children include the flexibility to test children at convenient times, the option to partition testing into multiple sessions, and enhanced comfort with both the tester (i.e., their caregiver versus an unfamiliar tester) and the environment (i.e., their own home versus a sound booth).

There are several important factors to consider with respect to implementing a remote testing experiment with children. Ambient noise levels are higher and more variable in homes relative to sound-treated rooms located in quiet laboratories. Children are more susceptible to the detrimental effects of competing background sounds than adults (reviewed by Leibold and Buss, 2019). Thus, ambient noise in the home may be particularly problematic for children. Another consideration for remote testing is variability in the adult supervision; whereas data collection in the laboratory usually involves 1–2 highly trained testers, a parent or caregiver is the primary tester for remote testing. Finally, consideration needs to be given to the hardware and connectivity requirements of the experiment. For example, level effects on the ability to benefit from interaural difference cues have been documented in the context of the MLD (Blauert, 1997), and hardware calibration is less precise when hardware varies across test sites.

The present experiment assessed the feasibility and reliability of using a 3AFC word recognition task for remote assessment of children's masked speech recognition and the BILD. Feasibility was evaluated by comparing data collected in participants' homes using participants' personal computers and headphones to data that were previously collected in a laboratory sound booth using calibrated equipment and high-quality circumaural headphones (Schneider *et al.*, 2018). Additional factors to be evaluated included how many participants were able to successfully perform the task in their own home, ambient noise levels in the home, and participant report of disruptions that occurred during testing. Reliability was assessed by evaluating the variability of BILD estimates across estimates obtained on different days.

## 2. Methods

### 2.1 Listeners

Participants were native English-speaking children from ages 6.7 to 17.6 years (*n* = *28*; *mean age = 10.7*, *stdev = 3.1*; 15 females) and adults ages 22.8 to 45.8 years (*n=*11; *mean age = 33.4*, *stdev = 9.1*; 7 females). All had normal hearing and negative history of ear infection within 30 days prior to the study by parent or self-report. Six of the adults were parents of child participants. Among child participants, most had a family member who also provided data, including siblings (*n* = 25) and/or parents (*n = 12*). Participants included prior research participants and new recruits, with approximately equal numbers of each (20 prior, 19 new).

### 2.2 Stimuli and equipment

Target stimuli were 25 sets of three monosyllabic words; each three-word set was composed of words that shared consonants and differed with respect to the central vowel (e.g., steak, stack, and stick; pea, pie, and paw). All words were within the expressive vocabularies of 5- to 6-year-old children in the United States (Storkel and Hoover, 2010). Targets were spoken by a female talker. The masker was a white noise, shaped to the long-term average spectrum of the targets. A compiled matlab script (MathWorks, Natick, MA) was used to present stimuli and collect listener responses. Participants downloaded software over the internet, ran it on a personal computer, and listened to stimuli using personal headphones. Prior to providing data, listeners were asked to set the sound output level on their computer at 50%, although they could adjust the level if it was judged to be uncomfortable. Pilot data using a range of commercially available hardware indicated that this setting resulted in a mean stimulus level of approximately 60 dB sound pressure level (SPL). Once set, participants were instructed not to adjust the volume throughout the duration of the experiment on all test days.

### 2.3 Procedure

Participants were provided with written instructions incorporating screenshots of critical elements (e.g., BILD program home screen) and a step-by-step checklist, which guided them through steps for downloading, installing, and running the experimental program. They were also provided with individualized instructions regarding the order of conditions, which were randomized for each participant. Caregivers of child participants were provided with general guidelines for supervising their child during data collection; they were asked to test in a quiet space, reduce background noise and distractions, take breaks, and test equipment themselves before beginning data collection. Prior to the first test session, listeners and/or parents' video-conferenced with the experimenter (Cisco Webex, Milpitas, CA), who obtained consent, reviewed the instructions, and answered questions. The second session occurred the day after the first session, and the third session occurred seven days after the first (i.e., Day 1, Day 2, and Day 7). Participants were encouraged to contact the experimenter *via* email with questions or difficulties carrying out the protocol.

The task was 3AFC word recognition. For each trial, illustrations corresponding to one of the three-word sets were shown on the screen. One of those words was presented over headphones, and participants were instructed to respond by clicking on the illustration associated with the word that they heard. Target words were presented either in-phase (*T*_0_) or out-of-phase (*T*π); the masker, when present, was in-phase (*M*_0_) across ears. Recognition of binaurally in-phase targets was also measured in quiet. The SRT was determined adaptively, and the stimulus condition was held constant within a threshold estimation run. Signal-to-noise ratio (SNR) was adapted using a two-down, one-up procedure with eight reversals per run; SRT was calculated as the average SNR at the last six reversals. SRTs in quiet were obtained using the same procedure, but with the amplitude of the noise masker set to zero. For each session (one on each day), seven runs were completed. Quiet SRTs were measured in a single run, completed at the beginning of testing each day. Masked SRTs in noise were measured in three blocks of two runs each; the order of conditions in each block (*M*_0_*T*_0_and *M*_0_*T*_π_) was randomized. The difference between average SRTs on each test day for the *M*_0_*T*_0_ and *M*_0_*T*_π_ conditions comprised estimates of the BILD for each participant.

In addition to completing the word recognition task, participants measured environmental noise level at the beginning and end of each run using the NIOSH SLM app (iOS users) or the Sound Meter and Noise Detector app (Android users). At the end of each run, they were also prompted to report any distractions that occurred during that run. Data were saved and managed using the Research Electronic Data Capture (REDCap; Harris *et al.*, 2009) platform hosted at Boys Town National Research Hospital (BTNRH). Participants were compensated $15 per hour. All procedures were approved by the Institutional Review Board at BTNRH.

### 2.4 Comparison laboratory data

Laboratory data utilized for comparison to the remote data collected in this study were previously reported by Schneider *et al.* (2018). Listeners in that study were 15 children, from 6.3 to 17.1 years of age (*mean age = 11.5*, *stdev = 3.0*; 11 females). These participants were different from those who participated in the remote study. All were native speakers of American English and had normal hearing at octave frequencies from 0.25 to 8 kHz, confirmed on the day of test. The experimental protocol was as described above, with the following exceptions. Laboratory testing took place in a sound-proof booth using standard laboratory equipment, including circumaural headphones (HD25, Sennheiser) and an external soundcard (Babyface, RME). The overall stimulus level was fixed at 60 dB SPL, and listeners completed three adaptive threshold estimation runs in each condition. In contrast to the remote data collection protocol, all laboratory data were collected in a single test session.

### 2.5 Statistical analysis

Statistics were computed in R (R Core Team, 2019). Pearson correlation and Welch's t-test were used to evaluate effects of child age and listener age group, respectively. Given the directional predictions associated with age, these tests are reported one-tailed. Reproducibility of SRTs was evaluated with a two-way consistency model of intraclass correlation (ICC) implemented using the irr package in R (Gamer *et al.*, 2019). When the ICC is 0.75–0.90, reliability is said to be “good,” and values >0.90 are said to indicate “excellent” reliability (Koo and Li, 2016).

## 3. Results

Most listeners who agreed to participate provided data in all conditions, according to the protocol. Only 2% of the desired data were missing, in most cases due to failure to upload to REDCap. Four participants (three children, one adult) erroneously repeated a condition, one child did not complete Day 7 data collection, and one child is missing the SRT in quiet for Day 2 due to reported software errors.

Noise measurements reported at the beginning and end of each run had a median value of 39.3 dB SPL (IQR: 34.2–41.8 dB SPL). Correlations between mean noise levels and SRTs in quiet were minimal and did not reach significance for any test day (*r* = 0.27, Day 1; *r* = –0.02, Day 2; *r* = 0.20, Day 7). Results were qualitatively similar when the analysis was restricted to just data from adult listeners. Participants reported distractions on 10% of runs. Of these, the most common factor cited was noise generated by other people in the vicinity of the listener (41%), followed by pets (21%), self-generated noise (e.g., sneezing, stomach growling, 11%), mechanical noise (e.g., fans, 11%), and difficulties associated with the test hardware (e.g., loss of internet connectivity, 2%). For the remaining cases (14%), the nature of the distraction was unspecified or attributed to multiple factors. The mean difference in SRTs for runs with and without reported distractions in quiet on Day 1 was −3.8 dB, indicating better SRTs on average for runs associated with distractions. The mean difference for masked SRTs were 1.0 dB for the *M*_0_*T*_0_ and 1.2 dB for *M*_0_*T*_π_, indicating very slightly poorer performance on average for trials with reported distractions. However, these differences are small compared to differences between conditions and between individuals.

Recall that SRTs in quiet were measured using the same procedures as masked recognition but reducing the masker amplitude to zero. For SRTs well below 0 dB, the reference level is determined by the 50% system sound level setting and any subsequent adjustments to ensure listener comfort (dB RE: comfortable). Mean SRTs in quiet on Day 1 were −35.1 dB RE: comfortable for children and −42.1 dB RE: comfortable for adults. This group difference was significant, indicating better performance for adults (*t*_15.8_ = 1.76, *p* = 0.049). In data for child listeners, there was a correlation between age and SRT in quiet (*r* = –0.32, *p* = 0.048) indicating better performance for older children. One rationale for measuring SRT in quiet was to determine the extent to which *masked* speech recognition might be limited by absolute audibility. The SRT in quiet was compared to the lowest masked SRT for each listener on each test day. The mean difference was 23.2 dB (*stdev* = 11.0 dB) for children and 24.4 dB (*stdev* = 9.0 dB) for adults. The difference between SRT in quiet and the minimum masked SRT was<6 dB in only 3% of cases (2 datasets for children and 1 for an adult). This result supports the conclusion that masked recognition was not appreciably limited by audibility.

**Fig. 1.**
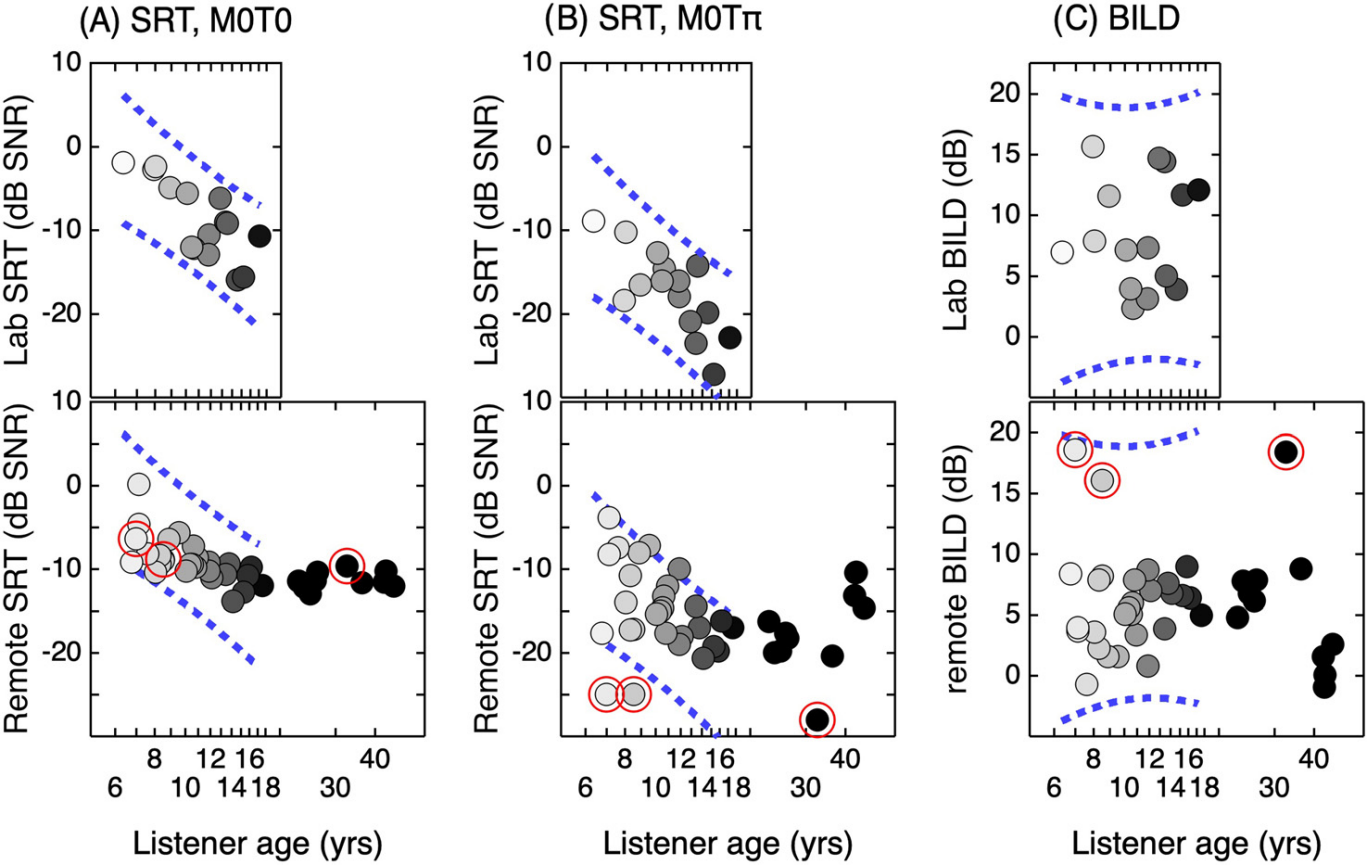
Results for laboratory testing (top row) and Day 1 of remote testing (bottom row), shown separately for SRTs in the M_0_T_0_ condition (column A), SRTs in the *M*_0_*T*_π_ condition (column B), and BILD (column C). Data are plotted as a function of listener age, which is also represented with symbol shading. Dotted lines indicate the 95% prediction interval for the line fit to laboratory data as a function of child age. Remote data for three listeners are highlighted with circles. These listeners were a parent and two children from the same family, all of whom listened using the same hardware; all three had unusually low SRTs in the *M*_0_*T*_π_ condition compared to other listeners, and as a result, unusually high BILDs.

Figure 1 shows masked speech recognition data collected in the laboratory and remote data from Day 1, plotted as a function of listener age. Laboratory data (top row) are considered first. In that dataset, SRTs improved with child age for both the *M*_0_*T*_0_ and *M*_0_*T*_π_ conditions (*r* = 0.74, *p* < 0.001; *r* = 0.77, *p* < 0.001). There was no evidence of a positive association between BILD with age (*r* = 0.09, *p* = 1.000). The dotted lines in Fig. 1 indicate the 95% prediction intervals around line fits to laboratory data as a function of child age. Prediction intervals for laboratory data are replicated in panels depicting remote data. Remote data from children fall within these bounds in all but four cases, all in the *M*_0_*T*_π_ condition: two SRTs were just above the upper bound, and two were below the lower bound.

Results obtained remotely are generally similar to those observed in the laboratory. For the *M*_0_*T*_0_ condition, mean SRTs were –8.9 and −11.3 dB SNR for children and adults, respectively; this difference between age groups was significant (*t*_36.8_ = 4.13, *p* < 0.001), and there was a significant reduction in SRT with increasing child age (*r* = –0.64, *p* < 0.001). For the *M*_0_*T*_π_ condition, mean SRTs were −15.0 and –17.1 dB SNR for children and adults, respectively; this group effect was not significant (*t*_18.6_ = 1.19, *p* = 0.125), and the trend for a reduction in SRT with increasing child age was likewise not significant (*r* = –0.31, *p* = 0.054). Visual inspection of data in the *M*_0_*T*_π_ condition reveals three data points that are low compared to other participants' data, as indicated with red circles. These three participants were all from the same family and used the same hardware for data collection. Their SRTs in the *M*_0_*T*_0_ condition were unremarkable, but SRTs in the *M*_0_*T*_π_ condition were 7.1–9.6 dB lower than other participants. Excluding the two child outliers resulted in a significant correlation between child age and SRT in the *M*_0_*T*_π_ condition (*r* = –0.57, *p* = 0.001). Mean BILDs were 5.8  and 6.1 dB for children and adults, respectively; groups were not significantly different (*t*_14.9_ = 0.14, *p* = 0.446), and the association between child age and BILD was not significant (*r* = –0.03, *p* = 0.56). Excluding the two child outliers resulted in a non-significant trend for a correlation between age and BILD (*r* = 0.32, *p* = 0.055). The pattern of results observed on Day 1 was representative of those observed on Day 2 and Day 7.

**Fig. 2.**
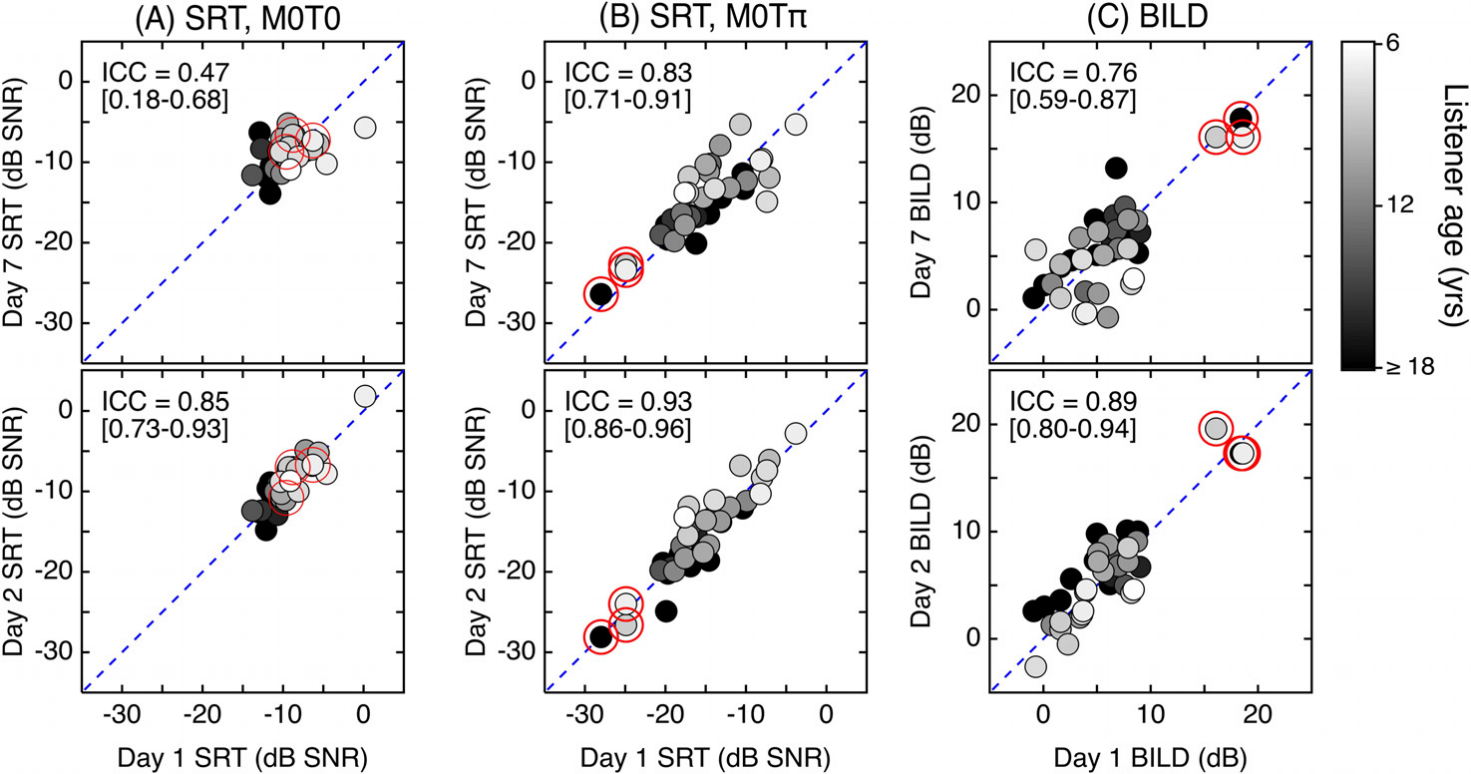
Association between SRTs at Day 1 compared to Day 2 and Day 7. Results are plotted separately for SRTs in the *M*_0_*T*_0_ condition (column A), SRTs in the *M*_0_*T*_π_ condition (column B), and estimates of the BILD (column C). Child age is indicated by symbol shading, as defined in the legend; adult data are shown with black fill. The interclass correlation and 95% confidence interval is indicated in the upper left corner of each panel, and the dashed diagonal line is included for reference. The three outlier listeners are highlighted with large circles.

The relationship between results obtained on Day 1 and subsequent test intervals is illustrated in Fig. 2. Based on the 95% confidence intervals (CIs) around each estimate of ICC, there was significantly greater consistency between Day 1 and Day 2 than between Day 1 and Day 7 for SRTs in the *M*_0_*T*_0_ and the *M*_0_*T*_π_ stimulus conditions, as well as for the BILD. For the M_0_T_0_ condition [Fig. 2(A)], this difference was pronounced, with ICC values of 0.85 (CI 0.72–0.92) and 0.47 (CI 0.18–0.68), respectively. The lower ICC for Day 1 and Day 7 appears to be due in part to the ∼6-dB improvement in SRT for the poorest-performing child (7.1 years). However, a significant difference is still observed when this child's data are omitted, and there is no evidence of a substantial improvement in group mean performance over time. For the *M*_0_*T*_π_ condition [Fig 2(B)], values of ICC were 0.93 (CI 0.86–0.96) and 0.83 (CI 0.71–0.91), respectively. For the BILD [Fig. 2(C)], values of ICC were 0.89 (CI 0.80–0.94) and 0.76 (CI 0.59–0.87), respectively. Greater consistency between Day 1 and Day 2 compared to Day 1 and Day 7 in all three outcome measures is also observed when analysis is restricted to child data and when data from the three outliers are omitted.

The ICC for SRTs in quiet (not shown) did not differ significantly for Day 1 and Day 2 (ICC = 0.57, CI 0.32–0.75) compared Day 1 and Day 7 (ICC = 0.46, 0.18–0.68). While not significant, the magnitude of this difference in ICC scores is comparable to that observed for the *M*_0_*T*_π_ and the BILD. Failure to observe a significant difference in the ICC for SRT in quiet could be due in part to the fact that quiet SRTs were estimated using one adaptive threshold run, whereas masked SRTs were estimated using three such runs.

Additional data collection was undertaken to better understand the particularly good SRTs in the *M*_0_*T*_π_ condition (and thus the particularly high BILDs) from the three outlier listeners highlighted in Figs. 1 and 2. The adult and one child (an 8-year-old) repeated data collection using their computer hardware (as previously), but with standard laboratory headphones (HD25, Sennheiser) instead of their personal headphones. Results obtained using the laboratory headphones resembled those from other listeners. For the *M*_0_*T*_π_ condition, average SRTs were −18.3 and −13.9 dB SNR with laboratory headphones, compared to −27.5 and −24.7 dB SNR with personal headphones. Average values of the BILD were 6.4 and 3.7 dB with laboratory headphones, compared to 17.8 and 17.3 dB with personal headphones. It is unclear what feature of this family's headphones is responsible for the marked difference in results.

## 4. Discussion

The purpose of this work was to assess the feasibility and reliability of using a 3AFC word recognition task for remote assessment of the BILD in children. Results support the general feasibility of BILD measurement in a remote setting but indicate more variability than observed for data obtained in the laboratory.

All participants were able to perform the task, providing a complete data set for Day 1 testing. Only 2% of data were missing across all three days of testing, with the majority attributable to REDCap upload failure. In addition, the minimum masked SRT was >6 dB above the SRT in quiet in 97% of cases, supporting the conclusion that masked recognition was not considerably limited by audibility. This finding is consistent with reports of low background noise levels measured before and after each run (*median* = 39.3 dB SPL) and the number of runs disrupted by environmental sources (10%). However, the use of personal hardware resulted in outlying data for at least one household, and a small but notable proportion of data were lost during remote data transfer.

Despite the limitations described above, remote SRTs (*M*_0_*T*_0_ and *M*_0_*T*_π_) and BILDs from children fell within the 95% prediction intervals of data collected in the laboratory setting in all but four cases, all of which occurred in the *M*_0_*T*_π_ condition. Two of these cases are associated with outlier data produced from the same household. In these two cases, follow-up testing using laboratory headphones produced results similar to those obtained in the laboratory. Nonetheless, remote BILD data obtained from children largely followed data patterns obtained in a laboratory setting.

Similar to the data of Koopmans *et al.* (2018), SRTs improved with increasing child age for both the *M*_0_*T*_0_ and *M*_0_*T*_π_ conditions. Koopmans *et al.* (2018) also reported an exponential increase in the BILD as a function of child age from 4 to 12 years of age. In contrast, neither the laboratory data nor the remote data of the present study indicate an age effect. However, participants in the present study (from 6 to 17 years) were older than the cohort studied by Koopmans *et al.* (2018). It is possible that inclusion of more and/or younger children in the present study could have revealed an age effect. Importantly, the similarity between data obtained remotely from the present study and previous data obtained from laboratory settings suggests that remote factors (e.g., ambient noise levels, untrained adult supervision versus trained staff) played little or no role in the outcomes observed here.

Comparison of masked SRTs and the BILD assessment for Day 1 and Day 2 testing indicates good reliability. In contrast, results from Day 1 and Day 7 were less consistent, particularly for the *M*_0_*T*_0_ condition. It is not clear how to account for greater consistency between masked SRT for estimates obtained on Day 1 and Day 2 compared to Day 1 and Day 7. Decreased consistency for the longer delay between test intervals could represent differences in listener strategy or differences in settings on the test computer (e.g., volume settings, despite participants being instructed maintain consistent volume settings for all testing), either of which could change over time. The latter possibility is inconsistent with the failure to observe reduced reliability between SRTs in quiet on Day 1 and Day 7 compared to Day 1 and Day 2, with the caveat that reliance on a single run for testing in quiet could reduce the power of this comparison.

While our results indicate that masked speech recognition and the BILD evaluated in a remote setting broadly replicate results obtained in the laboratory, some notable limitations related to remote testing emerged. First, outlying data resulted from the use of personal hardware. Although results from the present study suggest some degree of consistency when using a range of personal hardware configurations, the outlier data obtained from one family represent a notable exception to that general conclusion. As such, results from the present study support the use of rigorous procedures for identifying outliers when collecting data remotely using personal hardware. Another approach could be to utilize a pre-test prior to data collection to assess the hardware and ensure results in the expected range. Another limitation to the current study was the inability to control or monitor stimulus presentation levels on personal hardware. This limitation is particularly problematic for tasks and conditions that are known to be level-dependent, like the MLD (Blauert, 1997). Last, procedures for automatically uploading data in the current protocol resulted in data loss. This feature was intended to increase efficiency; however, approaches that verify active internet connection prior to upload may be preferable.

## 5. Conclusions

The goal of this work was to assess the feasibility and reliability of remote assessment of masked speech recognition and the BILD in children. Results demonstrate that remote assessment of these tasks in children is both feasible and generally reliable, though outlier responses did result in some cases, which appears to be due to the use of personal hardware. Supplying or specifying specific hardware (e.g., headphones) would likely improve reliability in a remote testing environment.
